# Targeting Th17 cells: a promising strategy to treat oral mucosal inflammatory diseases

**DOI:** 10.3389/fimmu.2023.1236856

**Published:** 2023-07-26

**Authors:** Ying Wang, Ningning Xue, Ziyuan Wang, Xin Zeng, Ning Ji, Qianming Chen

**Affiliations:** State Key Laboratory of Oral Diseases, National Clinical Research Center for Oral Diseases, Research Unit of Oral Carcinogenesis and Management, Chinese Academy of Medical Sciences, West China Hospital of Stomatology, Sichuan University, Chengdu, Sichuan, China

**Keywords:** T helper cells, Th17, oral inflammatory diseases, oral mucosa, periodontitis, oral mucosal immunity

## Abstract

With the improved quality of life, oral health is under increased pressure. Numerous common oral mucosal diseases, such as oral lichen planus(OLP) and gingivitis, are related to the destruction of the oral immune barrier. The cytokines secreted by T-helper 17 (Th17) cells are essential for maintaining oral immune homeostasis and play essential roles in immune surveillance. When antigens stimulate the epithelium, Th17 cells expand, differentiate, and generate inflammatory factors to recruit other lymphocytes, such as neutrophils, to clear the infection, which helps to maintain the integrity of the epithelial barrier. In contrast, excessive Th17/IL-17 axis reactions may cause autoimmune damage. Therefore, an in-depth understanding of the role of Th17 cells in oral mucosa may provide prospects for treating oral mucosal diseases. We reviewed the role of Th17 cells in various oral and skin mucosal systemic diseases with oral characteristics, and based on the findings of these reports, we emphasize that Th17 cellular response may be a critical factor in inflammatory diseases of the oral mucosa. In addition, we should pay attention to the role and relationship of “pathogenic Th17” and “non-pathogenic Th17” in oral mucosal diseases. We hope to provide a reference for Th17 cells as a potential therapeutic target for treating oral mucosal inflammatory disorders in the future.

## 1 Introduction

The immune environment can change with different tissues, and usually due to tissues adapting to different functional requirements (1). The concept of barrier immunity has existed for many years. But most studies of barrier immunity originated from intestine, lung, and skin (2, 3). These studies do not necessarily apply to oral immunity because of the tissue and site specificity of the oral cavity. The oral cavity is the environment that continuously experiences physical and microbial stimuli, with maintaining a high level of immunity (4). Numerous oral mucosal diseases are related to chronic inflammation (5–7), including OLP, oral leukoplakia, recurrent aphthous ulcers, and gingivitis. Oral mucosal inflammatory diseases usually lack effective etiological treatment (8–10). Furthermore, the treatment is often lengthy and accompanied by dental erosion and pain, which causes inconvenience to the patients (11, 12). Then researchers have been attempting to find effective therapeutic methods by understanding the pathogenesis (13, 14). However, the potential solutions have been very few thus far. Clinically, the treatment of oral mucosal inflammatory diseases mainly focuses on symptomatic treatment (15, 16). In recent years, there has been a new understanding of oral mucosal immunity, and it is suggested that mucosal inflammation may be related to an imbalance in immune homeostasis (17–19). T-helper 17 (Th17) cells play an important role in oral mucosal barrier immunity (19, 20).

**Figure 1:**
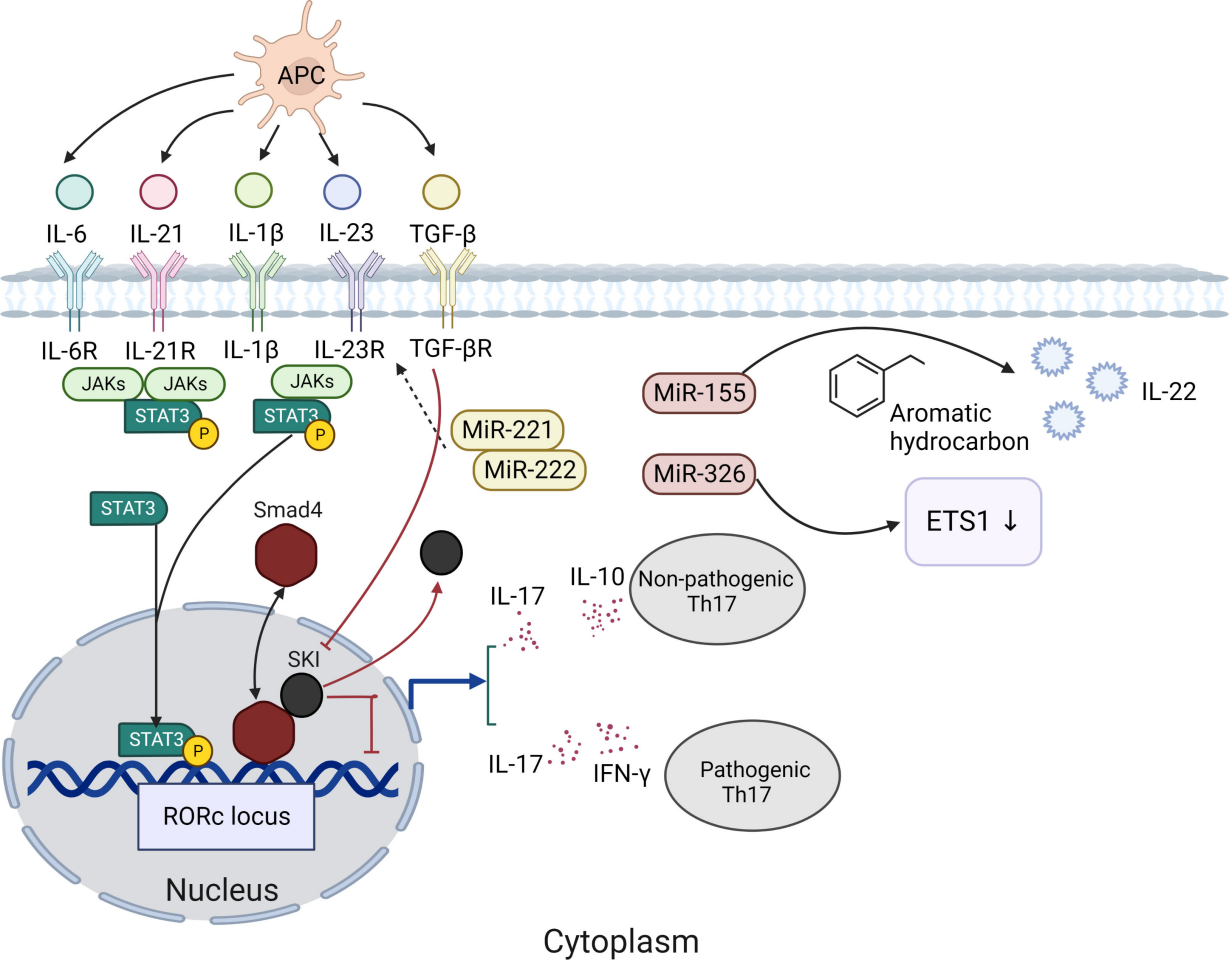
The significant factor in Th17 cell differentiation is the activation of signal transducer and STAT3. The binding of IL-6, IL-21, and IL-23 with receptors allows JAKs to phosphorylate the receptors, leading to recruitment and phosphorylation of STAT3. STAT3 subsequently undergoes dimerization and gets translocated to the nucleus to enhance the expression genes. STAT3 induces the expression of the ROR-γt. Th17 cells produce IL-17 and IL-10, also known as “non-pathogenic” Th17 cells. Th17 cells can produce IL-17 and IFN-γ and induce pathogenic inflammation while fighting pathogens; therefore, it is called “pathogenic” Th17 cells. TGF-β neutralized SMAD4-mediated inhibition without affecting SMAD4 binding to the Rorc site and promoted Th17 cell differentiation by reversing SKI/SMAD4.

Th17 cells are an important subset of CD4+ T cells, which can secrete a series of cytokines (including IL-17A, IL-17F, IL-21, IL-22, and GM-CSF). Th17 cells are associated with multiple mucosal inflammatory diseases (21, 22). And they can resist external pathogens, maintain barrier immunity, and undergo unique differentiation processes in different internal and external environments (23, 24). High-throughput sequencing revealed that Th17 cell development includes multiple positive and negative regulatory modules, illustrating the complexity of Th17 cell generation (21). The significant factor in Th17 cell differentiation is the activation of signal transducer and activator of transcription 3 (STAT3). The binding of IL-6, IL-21, and IL-23 with receptors allows Janus kinases(JAKs) to phosphorylate the receptors, leading to recruitment and phosphorylation of STAT3. STAT3 subsequently undergoes dimerization and gets translocated to the nucleus to enhance the expression genes (25) (**Figure 1**). STAT3 induces the expression of the transcription factor orphan nuclear receptor γt (ROR-γt). ROR-γt is thought to be a major regulator of Th17 cells (26, 27). The initial study suggested that the inducing factor of Th17 cells was IL-23, but naïve T cells did not express the IL-23 receptor, and other conditions were needed to induce the differentiation of primordial T cells into Th17 cells (28, 29). Subsequent studies have shown that transforming growth factor-β (TGF-β) and IL-6 can activate RORγt (30). RORγt can bind to transcription factors, such as STAT3, and promote the expression of IL-17A and IL-17F (31). Th17 cells produce IL-17 and IL-10, also known as “non-pathogenic” Th17 cells (32). Th17 cells can produce IL-17 and IFN-γ and induce pathogenic inflammation while fighting pathogens; therefore, it is called “pathogenic” Th17 cells (33). In other words, the presence of TGF-β may induce Th17 cells to exert different functions. However, some scholars believe the role of TGF-β in promoting Th17 cell differentiation is unclear (34, 35). SKI is a factor whose deregulation is closely associated with tumorigenesis, 1p36 deletion syndrome, and Shprintzen–Goldberg syndrome (36). At the same time, SKI is a transcription factor and inhibits H3K9 acetylation, RoRc expression and Th17 cell differentiation through SMAD4-dependent pathway. In contrast, TGF-β neutralized SMAD4-mediated inhibition without affecting SMAD4 binding to the Rorc site and promoted Th17 cell differentiation by reversing SKI/SMAD4 (37) (**Figure 1**). There is evidence that miRNAs can also regulate the proliferation and differentiation of Th17 cells. MiR-155 (**Figure 1**), which is expressed at high levels in both human and mouse Th17 cells, can increase the aromatic hydrocarbon receptor signaling pathway to promote the expression of IL-22 cytokine in Th17 cells (38). In addition, miR-326 is a positive regulator of Th17 differentiation, which acts by inhibiting ETS1 (Th cell differentiation inhibitor) (39). MiR-221 and miR-222 have been found to regulate Th17 immunity in the intestine by downregulating IL-23R and c-MAF and inhibiting IL-23-induced Th17 responses (40). Moreover, miR-221- and miR-222-deficient mice showed weaker protection against mucosal damage. These results suggest that miRNAs are also an important component of the Th17 cellular response. However, it has been suggested that the effects of miRNAs on Th17 function and differentiation cannot be distinguished (41, 42).

In addition, Th17 cells produce IL-21 and IL-23, which feedback to themselves and mediate the expansion and stability of Th17 cells (43). After differentiation, Th17 cells express chemokine C-C receptor (CCR)-6, which preferentially migrates to the mucosa and barrier sites (44). The IL-17 family includes IL-17A, IL-17B, IL-17C, IL-17D, IL-17E (also known as IL-25), and IL-17F (45). IL-17A and IL-17F show highly similarities and both play significant roles in the adaptive immune response, particularly against bacteria and fungi (46). They can stimulate the expression of pro-inflammatory cytokines and chemokines and the production of matrix metalloproteinases (MMP), thereby inducing an inflammatory response by recruiting immune cells to the site of infection. Whether Th17 or IL-17 is to be made the center of targeted therapy is still debated. When encountering external bacterial or fungal stimulation, Th17 cells secrete IL-17, which interacts with IL-22 to promote epithelial cells to secrete antibacterial peptides against pathogens (47). When the mucosal barrier is damaged, IL-17 promotes epithelial cells to secrete CCL-20, recruiting neutrophils to migrate to lesions, and plays a role in immune surveillance (48). Although initial research has been performed on the relationship between oral mucosal inflammation and Th17-type immunity, the specific mechanisms through which Th17 cells exert their immune effects remain unclear. Based on this, this study reviews the contribution of Th17 immunity in oral mucosal inflammatory diseases that have been discovered in recent years. We summarize the current research progress and identify the “two-sidedness” of these cells in oral mucosal immunity, a key factor in studying oral immune homeostasis.

## 2 Role of Th17 cells in mucosal immunity

### 2.1 Th17 cells in intestinal immunity

The intestinal and oral flora have similar complex immune mechanisms to maintain dynamic host-microbe balance, particularly Th17 immunity (49, 50). The intestine has the largest mucosal area and is rich in microorganisms (51). Th17 cells can be induced by bacteria and fungi, thus controlling important feedback pathways that inhibit bacterial and fungal over-proliferation. Th17 cells play an important role in barrier immune protection of the intestine (52). Among the T lymphocytes in the intestine, Th17 cells play an important role in secreting cytokines, such as IL-17A, IL-17F, and IL-22, promoting the production of mucin and AMP and activating immunoglobulin receptors to maintain the barrier function of the intestine (53). A significant Th17 immune response was observed in the intestine of germ-free mice, suggesting that the Th17 immune response is associated with the invasion of external pathogens (54). Furthermore, deficiency in CD4+ Th17 cells was observed in RORγt-deficient mice. They are sensitive to dextran sodium sulfate-induced colonic injury, suggesting that Th17 deficiency hinders the maintenance of intestinal immune homeostasis (55). These Th17 cells are present in the normal intestinal physiological environment and can be stable; hence, they are called “non-pathogenic Th17” in the intestine. However, they can be converted into “pathogenic Th17”. Conversion of “non-pathogenic Th17” to “pathogenic Th17” in the intestine demonstrates the plasticity of Th17 cells, a process that is dependent on IL-23. The deficiency of IL-23 reduces GM-CSF production by Th17 cells (56). B lymphocyte-inducible maturation protein 1 (BLIMP1) also drive Th17 “pathogenicity” because it enhances the expression of RORγt, STAT3, and histone acetyltransferase p300. IL-23 induces the production of BLIMP1 (57, 58). Without IL-23, Th17 cells are normal in number but fail to produce a pro-inflammatory Th17 cell response in the periphery (59). Thus, the availability of IL-23 in the intestinal environment may affect the balance between the beneficial and pathogenic responses of Th17 cells.

### 2.2 Th17 cells in pulmonic mucosal immunity

CD4+ T cells play an important role in lung diseases (24). For example, CD4+ T-cell deficiency in patients with AIDS increases the likelihood of lung infection. And Th17-type immune responses should be mentioned in inflammatory lung disorders. Cellular receptors mediate Th17-type immune responses, such as IL-17A, IL-17F, IL-21, GM-CSF, and IL-22, and are expressed in lung tissues, which underlines the possibility that Th17 cells mediate protective responses and pathological inflammation in the lung mucosa (60). In recent years, Th17 cells have been found to be involved in numerous acute and chronic inflammatory conditions in the lung (61, 62). In asthma, a class of refractory chronic inflammatory conditions, the conversion of Treg cells to Th17 cells is observed in mice. The external cell signaling regulatory kinase, IL-6, and STAT3 pathways jointly drive IL-17 expression (63). Another study found that mice lacking IL-17A receptors reduced Th1 immune-dependent infections, such as tuberculosis, but were unable to fight pulmonary *Klebsiella pneumoniae* infection because these mice had a reduced ability to recruit granulocytes to clear the infection (64, 65). The production of IL-17 was also required. At the same time, the regulatory role of miRNAs in Th17 cells in lung inflammation cannot be ignored. It has been found that miRNA-22 can control the activation of antigen-presenting cells and Th17 immune response by stimulating histone deacetylase HDAC4 (66). These results suggest that Th17 cells are important for protective responses in the lung mucosa, especially against pathogens. However, Th17 response is not always beneficial in the pulmonary mucosa. Chronic obstructive pulmonary disease is associated with abnormal microbial colonization, a process that exacerbates the Th17 response and inflammatory damage (67, 68).

## 3 Th17 cells in oral mucosal immunity

### 3.1 The barrier-protective role of oral Th17 cells

**Figure 2:**
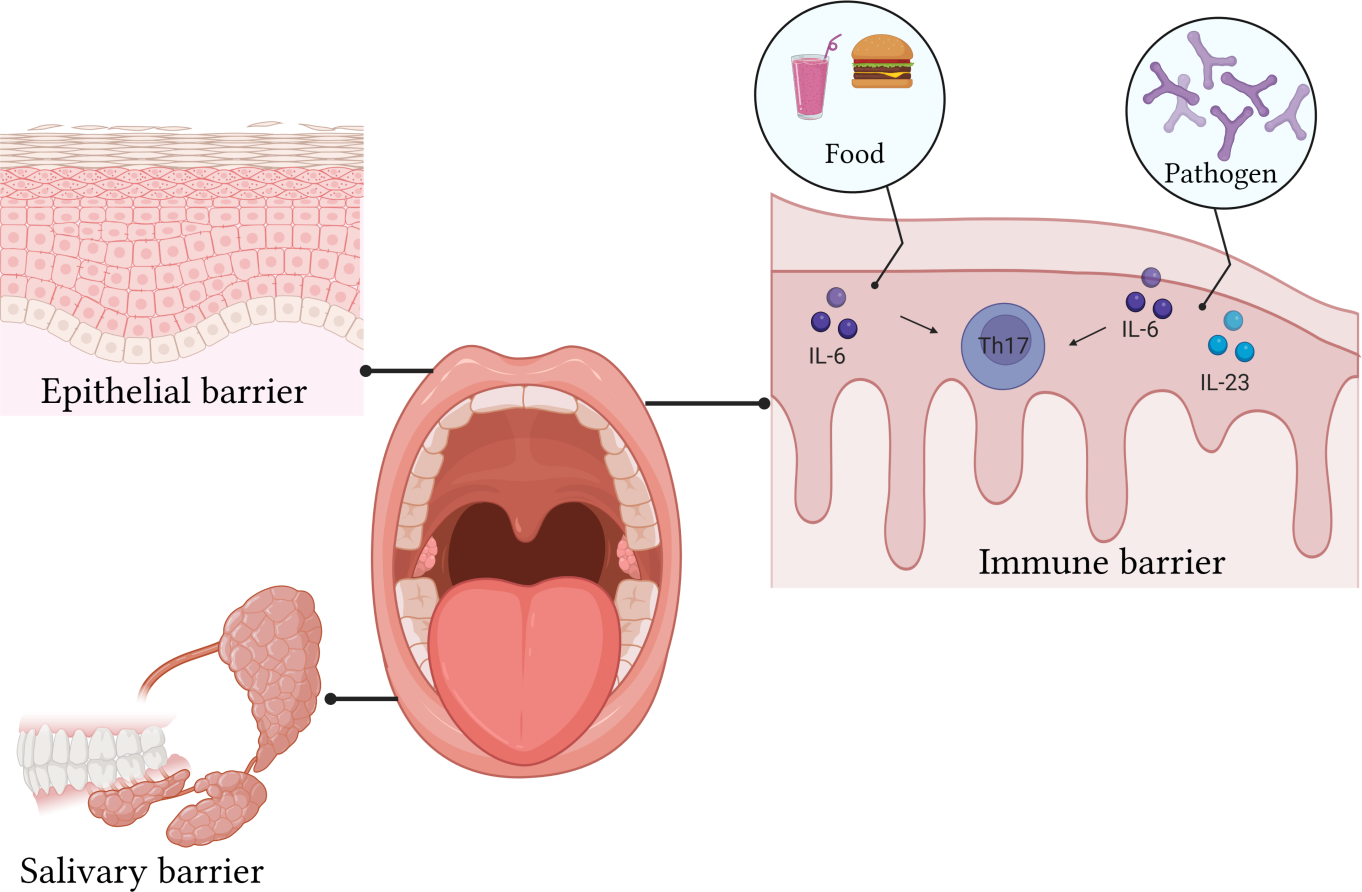
The epithelial layer is in direct contact with the external environment and is the first line of defense. The intact mucosal epithelium is a natural physiological barrier that prevents pathogens from entering deep tissues. The oral epithelium consists of various lymphocytes.

The oral mucosa is divided into an epithelial layer, lamina propria, submucosa, and basement membrane (69). The epithelial layer is in direct contact with the external environment and is the first line of defense (**Figure 2**). According to its location, it can be divided into keratinized and non-keratinized stratified squamous epithelium (70, 71). Additionally, many lymphocytes and plasma cells are present in the epithelial layer (72). However, these are transient cells that disappear with a reduction in inflammation. The lamina propria is a dense connective tissue that can support and nourish the epithelial layer (73). The submucosa contains blood vessels, nerves, and adipose tissue, providing nutrition and support to the lamina propria (74). The basement membrane is a collagen fiber complex that acts as a “bridge” between the connective tissues and epithelium. The intact mucosal epithelium is a natural physiological barrier that prevents pathogens from entering deep tissues (**Figure 2**). The immune barrier is related to host response. The oral epithelium consists of various lymphocytes (19). These lymphocytes are mainly present in the lamina propria.

The gastrointestinal tract and skin often elicit specific immune responses at the barrier owing to continuous contact with the external environment (75). As an important part of this barrier, the immune mechanism of the oral mucosa has received increasing attention in recent years. In addition, oral cavity is the first site of contact with external stimulation during eating or breathing. The uniqueness of the oral mucosa is that damage can be repaired quickly (20). Another unique feature is that the oral cavity is constantly stimulated by machinery such as mastication. These characteristics differ from those of the other barriers in the body.

Although the study of microorganisms has focused more on gut microbes, the oral cavity possesses a very diverse flora (76). Bacteria are the “mainstay” of oral immunity, but fungi also have an important role. Symbiotic flora is an important driver of barrier immunity. The differentiation of Th17 cells in the oral mucosa caused by microorganisms does not cause a disorder of the mucosal immune system but is very important for the protection of oral immunity (77). The protective mechanism of Th17 cells is different from that in other parts of the mucosa. The development of Th17 cells in the intestinal mucosa depends on the participation of IL-1, which is not required in the oral mucosa. In healthy oral mucosa, mechanical stimulation, such as chewing, can cause the accumulation of IL-6 in the epithelium, leading to the differentiation of Th17 cells and the production of IL-17 (20). When immune homeostasis is disrupted, oral bacteria, IL-6, and IL-23 in the oral mucosa jointly mediate the differentiation of Th17 cells (78).

### 3.2 Oral mucosal macular diseases and Th17 cells

OLP is a common chronic inflammatory disease of oral mucosa (79). Its prevalence rate is approximately 0.1–4%, which is second only to recurrent aphthous ulcers (RAU). The World Health Organization defines OLP as a precancerous lesion. However, the etiology of OLP is unclear (80). Numerous lymphocytes with dense zonal infiltration can be observed in the epithelial lamina propria of patients with OLP. Therefore, OLP may be associated with immune factors. The IFN-γ levels of OLP patients are upregulated, while the IL-4 levels are downregulated, and cellular immunity is enhanced, which makes them susceptible to OLP (81). With a further understanding of oral mucosal barrier immunity, the role of Th17 cells in OLP is being recognized. Th17 responses in OLP mainly focuses on the tissues of clinical patients. Saliva of patients with OLP was extracted for ELISA and flow cytometry analysis (82). It was found that IL-17 content in OLP patients was significantly higher than that in normal individuals, indicating that Th17 cells may play a role in the oral mucosal immunity of patients with OLP.

An increase in IL-17 and IL-23 levels was observed in the pathological tissues of patients with OLP (83). CD4+T cells in the peripheral blood of patients with OLP were extracted and stimulated with IL-23. The proportion of CD4+IL17+ cells increased, which was attributed to the increased proportion of Th17 cells. It was speculated that the IL-23/IL-17 axis and Th17 cells are related to the local immunity of OLP. In another study, it was found that the proportion of Th17 cells in erosive OLP tissue increased, whereas the proportion of Th2 cells in reticular OLP tissue increased significantly (84). RORγt, which has been recognized as an important transcription factor in Th17 cell differentiation, has also been detected in reticular and erosive OLP. However, there was no significant difference in RORγt expression between the two types of OLP (85). In a study by Javvadi (86), Regulatory T cells (Tregs) and Th17 cells were simultaneously studied in patients with OLP. They found that Th17 cells decreased and Tregs increased in OLP tissues, which was different from previous literature. However, in the author’s opinion, as immunosuppressive cells, the increase in Tregs indicated that the body exerts a high level of immune regulation in OLP lesions, which proved the particularity of local immunity in OLP patients to some extent. However, owing to the lack of recognized animal models, research on Th17 cells in OLP lacks effective animal experiments. The detection of clinical samples has shown that Th17 cells play an important role in the pathogenesis of OLP, and more investments in animal models are needed.

However, Th17 cells lack research on other oral mucosal macular diseases, such as oral leukoplakia, oral erythroplakia, and oral submucous fibrosis.

### 3.3 Gingivitis/periodontitis and Th17 cells

**Figure 3:**
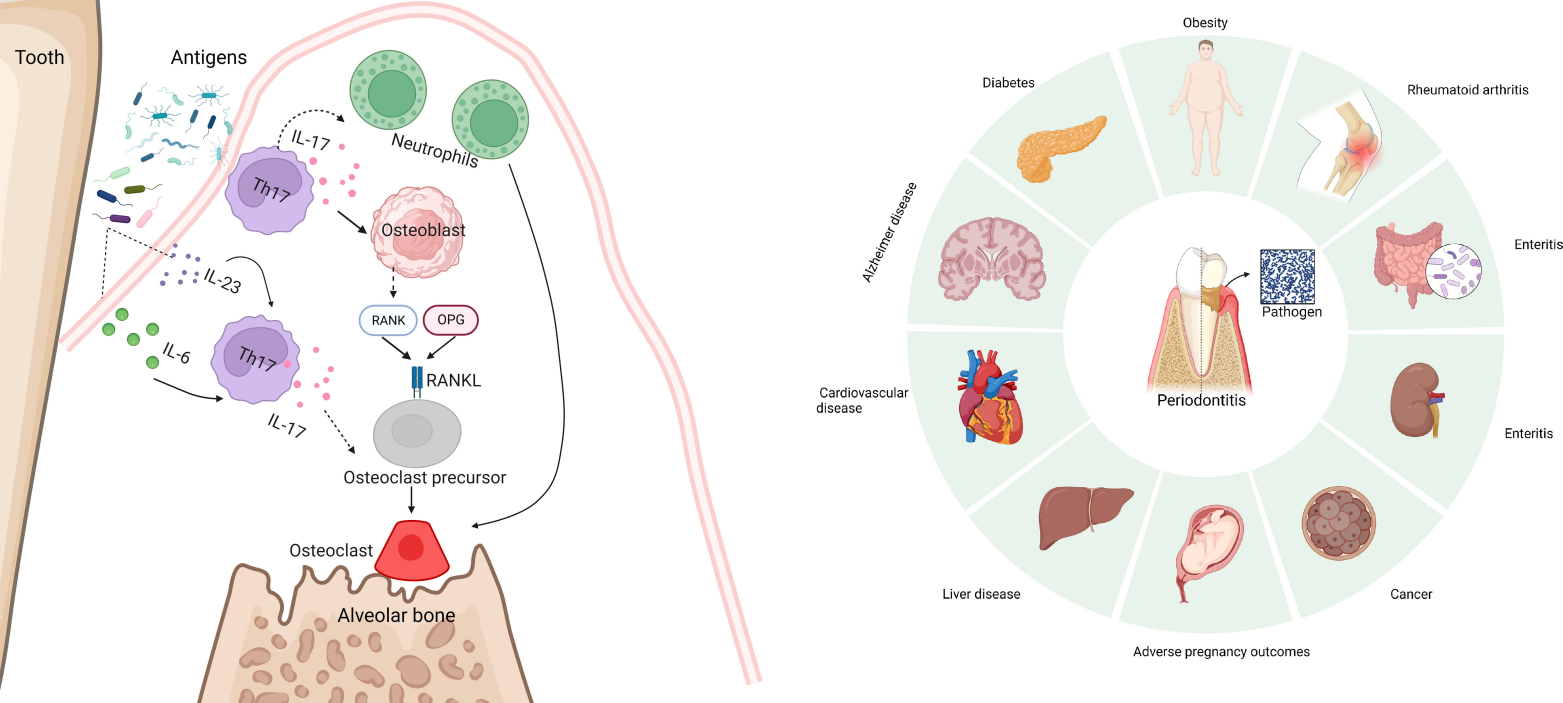
Periodontitis is one of the most common infectious diseases and is associated with various systemic diseases, including diabetes, metabolic syndrome, obesity, eating disorders, liver disease, cardiovascular disease, Alzheimer disease, rheumatoid arthritis, adverse pregnancy outcomes, and cancer. IL-17 can be directly or briefly involved in periodontal bone loss mediated by the Rank/Rankl pathway. And excessive accumulation of IL-17 aggravates bone loss in periodontitis.

Periodontitis is one of the most common infectious diseases and is associated with various systemic diseases (**Figure 3**) (87, 88). Gingivitis is the earliest and the most common manifestation of periodontitis. During gingivitis, bacteria cause tissue infection, interact with abnormal host immune responses and the microenvironment, and eventually cause periodontitis (89). T cell immunity plays a vital role in both the normal and inflammatory oral mucosa (5). Researchers have found that Th17 cells play a very important role in the occurrence and development of gingivitis and periodontitis.

Many bacterial groups are present in the mouth (90). A healthy mucosa can play an immunomodulatory role and form a dynamic balance with microorganisms. Once there is external stimulation, the balance is disrupted, and gingival inflammation appears. However, it is still debated whether Th17 cells and IL-17 play a promoting or inhibiting role in the occurrence and development of periodontitis (91, 92). IL-17 plays an immune surveillance role and has an anti-infection effect. When gingiva is stimulated by microorganisms, IL-17 recruits neutrophils to fight pathogen invasion (93). However, this is also a potential mechanism for inflammation-induced tissue destruction.

IL-17+ and Foxp3+ cells can transform into each other *in vitro* and are present in gingival tissue. When gingivitis occurs, IL-17 +/Foxp3- T cells are significantly increased, indicating that the process of mutual transformation exists in the body, and the downregulation of Treg proportion might be a probable reason for the decline in immunosuppression and inflammation (94). This possibility was verified in a study of gingivitis during pregnancy (95). Low levels of Tregs and high levels of Th17 were detected in the gingival tissue and cervical lymph nodes of pregnant mice during the progression of periodontitis. These studies highlight the important role of Th17/Treg balance in gingivitis and periodontitis.

In addition to its role in mucosal immunity, Th17 cells can cause bone destruction during periodontitis. IL-17 can be directly or briefly involved in periodontal bone loss mediated by the Rank/Rankl pathway (96) (**Figure 3**). Some studies have found that excessive accumulation of IL-17 aggravates bone loss in periodontitis (97). Local anti-IL-17A neutralizing antibody treatment reduced the disease course. IL-17A neutralizing antibodies can reduce the pathogenicity of oral flora in diabetic mice (98). When the oral microorganisms of the donor treated with IL-17 antibody were implanted into sterile mice, neutrophil recruitment was reduced, and IL-6 and RANKL of gingival tissues reduced; thus, bone absorption was reduced. However, in an animal model, inhibition of IL-17 led to periodontitis (99). As an important cytokine that plays an important role in immune surveillance, the lack of IL-17 does not seem to prevent periodontitis. An experiment conducted on a population found that the impaired development of Th17 cells did not cause a significant periodontitis phenotype (20, 94). However, oral endocrine IL-17 levels in patients with periodontitis increased. These results indicated that the role of Th17 cells and IL-17 in gingivitis/periodontitis cannot be evaluated simply by “promoting” or “inhibiting,” but could be studied as a link to mucosal immunity. The potential role of Th17 cells as a target for the treatment of gingivitis cannot be ruled out.

In addition, the interaction between Th17 cells and the microbiome cannot be overlooked (100). Excessive IL-17 inflammatory response and dysbiosis coexist in the chronic periodontitis setting. The LAD-1 periodontitis model demonstrated that an increased IL-17 response can cause an abnormal colonization of oral pathogenic flora (101). Furthermore, some studies have found that IL-17 or IL-23 inhibition could reverse dysbiosis to some extent, suggesting the contribution of excessive IL-17 inflammation to periodontitis (102–104). Periodontal pathogenic bacteria increased with increasing oral inflammation, and the above results demonstrate that IL-17 inflammatory mechanisms might be a key component of the dysregulated microbial-host balance in gingivitis/periodontitis. However, current studies are limited to animal models. The effects of IL-17 on dysbiosis are yet to be elucidated.

### 3.4 Oral candidiasis and Th17 cells

Fungal infection is a global clinical problem, and its incidence is continuously increasing (105). Among the symbiotic flora of human beings, the most common is *Candida albicans*, which can colonize the gastrointestinal tract, skin, urogenital system, and mouth (106, 107). Although some newborns experience thrush, the symbiotic state does not cause host damage (108). Once this balance is disrupted, it causes different degrees of host damage. The most common manifestation of fungal infections is inflammation of the mucous membrane and skin (109). If the fungus reaches important internal organs, it will be life-threatening. Although some healthy people carry *C. albicans*, mucosal immune disorders or fungal virulence enhancement can cause disease (110). Host factors play an important role in candidiasis pathogenesis. In adults, the incidence of oral candidiasis is higher in patients undergoing surgery, radiotherapy, or Sjogren’s syndrome than that in normal people (111).

The incidence of oral candidiasis in HIV patients is significantly higher than that in uninfected people (112). Therefore, it was suspected that the host factor for oral candidiasis might be the lack of CD4+T cells. In addition, the prevalence of *C. albicans* in patients with genetic or acquired immunodeficiency syndrome is increasing, indicating that CD4+T cells are essential to resist *C. albicans* infection (113, 114). Researchers have focused on Th17 cells at the start (115). The sensitivity of mice lacking IL-12p35 to oral candida was lower than that of mice lacking IL-23p19, IL-17RA, or IL-17RC, which indicated that *C. albicans* mainly mediated the Th17 pathway rather than Th1 (116). Some patients with immunodeficiency syndrome lack Th17 immunity, such as STAT3 loss-of-function (LOF) mutations (117), STAT1 gain-of-function mutations (118), bi-allelic RORC mutations (119), or CARD9 LOF mutations (120), showing a strongly increased risk of developing chronic metabolic syndrome. *In vitro* studies in human cells have shown that *C. albicans* mycelia induce the production of IL-23, a cytokine that drives the expansion and function of Th17 cells (121).

**Figure 4:**
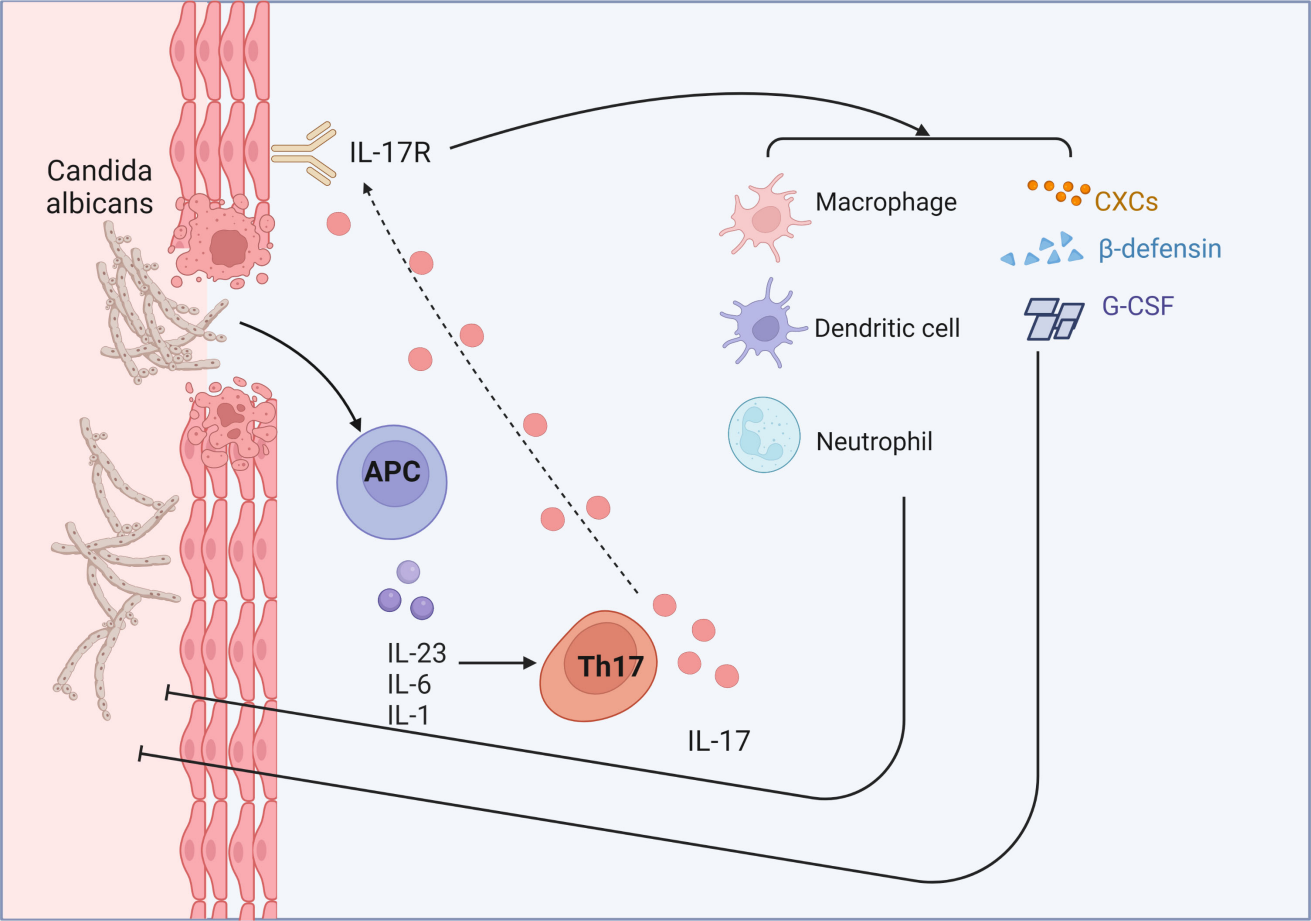
IL-17 relies on neutrophils, macrophages, and dendritic cells to kill fungi. IL-17 stimulates neutrophil-recruiting CXC chemokines (CXCL1, CXCL2 and CXCL5) and G-CSF, and helps the mucosa to fight the fungus. Furthermore, IL-17 is also a potent inducer of β-defensins.

The effect of Th17 cells on *C. albicans* depends mainly on IL-17(**Figure 4**). Studies have shown that mice lacking IL-17 receptor or its key downstream ACT1 are very sensitive to oropharyngeal candidiasis (122). In addition, patients with genetic mutations in the IL-17 receptor signaling pathway or high IgE syndrome (123) are prone to chronic mucocutaneous candidiasis, indicating that IL-17 has a certain immune effect on *C. albicans*. When the pathogen comes in contact with the epithelium, IL-17 relies on neutrophils, macrophages, and dendritic cells to kill fungi through oxidative (reactive oxygen species) and non-oxidative (hydrolases and antimicrobial peptides) pathways (32). Therefore, the production of IL-17 leads to a series of immune responses against *C. albicans*. IL-17 stimulates neutrophil-recruiting CXC chemokines(CXCL1, CXCL2 and CXCL5) and G-CSF, and helps the mucosa to fight the fungus (124, 125). Mice lacking the CXCR2 are more susceptible to candida albicans infection (126). Mice lacking neutrophils also exhibit candida susceptibility, and neutrophils can also exert anti-candida effects *in vitro* (127). IL-17 is also a potent inducer of β-defensins (BDs), and mice without BD3 has downregulated antifungal immunity (128). Treg also plays a significant role (129). On the one hand, it suppresses excessive inflammation induced by Th17. On the one hand, Treg enhances Th17 activity by depleting IL-2.

### 3.5 Oral mucosal ulcer diseases

RAU is the most common oral mucosal ulcer disease (130, 131). The immunological etiology of RAU is mainly cellular immunity. Patients show decreased cellular immune function and an imbalance in T-cell subsets. However, antinuclear antibodies, which are common in autoimmune diseases, have not yet been detected in humans. Thus, the immune response is only one of the possibilities for RAU treatment. Early studies found a dominant role of Th1 cell in the pathogenesis of RAU, especially the dysregulation of T cell ratio due to the conversion of Th2 cell to Th1 cell (132, 133). Due to the development of second-generation high-throughput sequencing and the discovery of Th17 cell, IL-17 is found in some clinical samples such as gingival sulcus fluid and peripheral serum (134, 135). These results suggest a possible role of Th17 cell in the pathogenesis of RAU and has great potential for research.

## 4 Th17 cell is a therapeutic target for skin-mucosal immune diseases

Studies have identified Th17 cells and IL-17 as potential therapeutic targets for skin mucosal immune diseases (136). However, recognized effective targeted Th17 therapy has been directed toward psoriasis. IL-17 accumulation reduces lipoprotein transport, thereby causing serious complications, such as vascular sclerosis complicated by atherosclerosis. Anti-IL-17 therapy can significantly reduce this risk (137, 138). In addition, IL-17-block therapy has resulted in breakthroughs in the study of multiple sclerosis (139). However, targeted therapy may not have a significant effect on rheumatoid arthritis (140). Targeted therapy of Th17 cells does not play a beneficial role. Another study suggested to focus on the switch key of Th17 cells: IL-23, the results of which remain to be further considered (141).

Lichen planus can invade the mouth as well as the skin and mucosa of other parts (142). A clinical study showed that the use of Anti-IL-17A monoclonal antibodies or inhibition of the Th17/Tc17 axis could alleviate lesions in patients (143), which indicated that IL-17 is a potential entry point for OLP treatment. Uveitis is a serious complication in patients with Behçet’s disease and is associated with autoimmune disorders. Studies have shown that the activation of the IL-23/IL-17 pathway is related to the activation and proliferation of pathogenic Th17 cells (144, 145). Drugs targeting the IL-23/IL-17 pathway have been shown to alleviate autoimmune uveitis (146). In addition, studies have found that the increase in Th17 reaction mediated by IL-21 and the inhibition of Tregs are closely related to the severity of Behçet’s disease (147, 148). It is unclear whether RAU is associated with Th17 cells. As a related disease, these studies provide a reference for the clinical treatment of RAU. Pemphigus is another serious autoimmune disease of the chronic mucosal skin autoimmune disease (149). The most common of them is pemphigus vulgaris, which is characterized by bullae in the oral mucosa. There are limited studies on Th17 cells in oral mucosal lesions of pemphigus, but elevated levels of IL-23 and IL-17 have been observed in the serum and skin lesions of patients with pemphigus (150, 151). Further studies on skin samples from patients with pemphigus revealed that the immune response was mainly mediated by IL-17A-related factors. In addition, another independent CD4+T cell subgroup, Tfh, can induce B cells to produce anti-Dsg-specific antibodies (152). These findings provide a new direction for the treatment of oral mucosal lesions associated with pemphigus. Many drugs, inhibitors or neutralizing antibodies targeting Th17 were used to treat skin-mucosal inflammatory diseases, and some of the completed and ongoing clinical trials are listed in **Table 1**, including, for example, IL-17, IL-6, and IL-23.

**Table 1:** Th17-targeted drugs in clinical trials.

Targeting Drug		Method of delivery	Disease Model	Phase	NCT code	Ref.
IL-17	MSB0010841	s.c.	Psoriasis	I	NCT02156466	(153)
	Anti-IL-17A Humanized Monoclonal Antibody	s.c.	Psoriasis	II	NCT05604898	(154)
	608 Q2W	s.c.	Psoriasis	III	NCT05536726	(154)
	JS005	s.c.	Psoriasis	Ib/II	NCT05344248	(155)
	BCD-085	s.c.	Psoriasis	II	NCT02762994	(156)
	LY2439821	s.c.	Psoriasis	II	NCT01107457	(157)
	AZD0284	p.o.	Psoriasis	I	NCT03310320	(158)
	CJM112	s.c.	Psoriasis	I	NCT01828086	(159)
	SHR-1314	s.c.	Psoriasis	II	NCT04121143	(160)
	Ixekizumab	s.c.	Lichen Planus	I	NCT05030415	(161)
IL-6	Adalimumab	s.c.	Psoriasis	Not Applicable	NCT01320293	(162)
	Tocilizumab	p.o.	Behçet’s disease	II	NCT03554161	(163)
	TA-650	i.v.	Behçet’s disease	III	NCT01532570	(164)
IL-23	LY2525623	i.v.	Psoriasis	II	NCT01018810	(165)
	Risankizumab	s.c.	Psoriasis	IV	NCT04630652	(166)
	Mirikizumab	s.c.	Psoriasis	III	NCT03535194	(167)
	CNTO 1959	s.c.	Palmoplantar Pustulosis	II	NCT01845987	(168)
	LY3074828	s.c.	Psoriasis	III	NCT03556202	(169)
	CNTO 1275	s.c.	Psoriasis	III	NCT00267969	(170)
	Ustekinumab	s.c.	Behçet’s disease	II	NCT02648581	(171)

## 5 Concluding remarks

The mucosa is the “protective layer” of the body’s immune system, is constantly exposed to external stimuli, and has a high and rapid immune response. To recognize stimuli and protect the body promptly, the normal mucosa maintains a relatively stable state and dynamic immunity balance. T-cell immunity plays an important role in the intestinal mucosa, lung mucosa, and oral mucosa. Th17 cells are a newly discovered subpopulation of CD4+ T cells, and its discovery explains part of the disease pathology that could not be elucidated by the classical Th1 and Th2 signaling pathways but also adds complexity to the study of mucosal immunity.

Oral mucosal diseases are usually caused by multiple factors, especially gingivitis, OLP, and RAU, which are highly prevalent in the population. The exact cause of most oral mucosal diseases cannot be determined, making clinical diagnosis and treatment difficult. With the advancements in immunology and further exploration of oral immunity using emerging tools, the evidence of oral mucosal barrier immune dysfunction and related diseases has changed, with Th17 cells predominating. There are many clinical treatments, including pharmacotherapy, intraoral laser therapy, and local closure therapy, to relieve the pain and discomfort caused by erosive mucosal lesions. However, the underlying mechanisms have not been fully elucidated. In this study, we reviewed the important roles of oral mucosal barrier immunity and Th17 cells in various oral mucosal diseases. In addition, we briefly summarized the progress in our understanding of systemic skin mucosal inflammatory diseases characterized by oral mucosa, which provides a reference for the treatment of these diseases. However, current research on Th17 cells is not completely clear. For example, although Th17 cells are classified as “pathogenic” and “non-pathogenic”, there seems to be no clear boundary for distinction. In addition, there are no studies on the type of Th17 cell that causes oral mucosal disease. Although many studies have shown that neutralizing Th17 or IL-17 antibodies can alleviate various autoimmune inflammatory diseases, IL-17 plays an important role in the treatment of the oral mucosa. However, an important role of IL-17 is immune surveillance, especially in the oral mucosa. The simple use of IL-17 antagonists is not a “panacea”. Does this affect the Th17/IL-17-mediated protective response, such as recruitment of neutrophils to fight infection? However, Th17 is a potential immunotherapeutic target in oral mucosal inflammation and deserves further investigation.

## Author contributions

YW drafted the manuscript. NX and ZW edited the manuscript. NJ and QC supervised the work and edited the manuscript. All authors contributed to the article and approved it for publication.
